# Effects of Repeated Low-Level Red-Light Therapy on Subfoveal Choroidal Thickness in Children with Myopia: A Systematic Review and Meta-Analysis

**DOI:** 10.3390/jcm15207862

**Published:** 2026-10-12

**Authors:** Yu-Yen Chen, Hsin-Chien Yen, Kuo-Hua Juan, Li-Ju Lin, Yun-Ju Lai, Yung-Feng Yen, Pesus Chou

**Affiliations:** 1 Department of Ophthalmology, Taichung Veterans General Hospital, Taichung 407, Taiwan; yuyenchen.phd@gmail.com (Y.-Y.C.); kohuajuan@vghtc.gov.tw (K.-H.J.); llju@vghtc.gov.tw (L.-J.L.); 2 Doctoral Program in Translational Medicine, National Chung Hsing University, Taichung 402, Taiwan; 3 School of Medicine, National Yang Ming Chiao Tung University, Taipei 112, Taiwan; lailai841081@yahoo.com.tw; 4 Program of Biomedical Informatics and Data Sciences, School of Medicine, Johns Hopkins University, Baltimore, MD 21218, USA; 5 School of Medicine, Chung Shan Medical University, Taichung 402, Taiwan; 6 Children’s Medical Center, Taichung Veterans General Hospital, Taichung 407, Taiwan; 7 Division of Endocrinology and Metabolism, Department of Internal Medicine, Puli Branch of Taichung Veterans General Hospital, Nantou 545, Taiwan; 8 Department of Health Care Management, National Taipei University of Nursing and Health Sciences, Taipei 112, Taiwan; 9 Section of Infectious Diseases, Taipei City Hospital, Heping Fuyou Branch, Taipei 100, Taiwan; 10 Institute of Public Health, National Yang Ming Chiao Tung University, Taipei 112, Taiwan

**Keywords:** repeated low-level red-light, myopia, premyopia, choroidal thickness

## Abstract

**Background/Objectives:** Repeated low-level red-light (RLRL) therapy has recently emerged as a promising treatment for childhood myopia. Choroidal thickening has been proposed as a key biological mechanism underlying its therapeutic efficacy; however, evidence regarding its effects on subfoveal choroidal thickness (SFCT), particularly in children with premyopia, remains limited. This systematic review and meta-analysis aimed to comprehensively evaluate the effects of RLRL therapy on SFCT in pediatric patients with myopia and premyopia. **Methods:** PubMed, Embase, and the Cochrane Central Register of Controlled Trials were systematically searched from database inception through 29 September 2026. Randomized controlled trials comparing RLRL therapy with controls in participants younger than 18 years were included. The primary outcome was the change in SFCT at 1, 3, 6, 9, and 12 months. Mean differences (MDs) with 95% confidence intervals (CIs) were pooled using random-effects models. Prespecified subgroup analyses were conducted according to refractive status. **Results:** Thirteen randomized controlled trials comprising 1974 children were included. In children with myopia, RLRL therapy was associated with significantly greater increases in SFCT than the control across all follow-up time points, with the greatest mean difference observed at 9 months (MD = 37.01 μm, 95% CI, 30.48–43.54). In children with premyopia, RLRL therapy also induced significant SFCT thickening from 1 to 9 months. Sensitivity analyses confirmed the robustness of the findings, and no evidence of significant publication bias was identified. **Conclusions:** These findings demonstrate consistent choroidal thickening following RLRL therapy in children with myopia and premyopia and support further investigation into the potential role of choroidal changes in myopia control.

## 1. Introduction

The incidence and prevalence of myopia have increased dramatically across generations worldwide. In East Asia, the prevalence of myopia among 15-year-old adolescents has reached approximately 69%, while a large cross-sectional study from Southern California reported a prevalence of 41.9% among children [1,2]. Myopic maculopathy is one of the leading causes of irreversible visual impairment in highly myopic individuals, and the risk of retinal detachment is estimated to be approximately 20-fold greater than that in emmetropic eyes [3,4]. In addition, high myopia is associated with increased risks of glaucoma and earlier cataract development [3]. Because the pathological changes associated with high myopia are largely established during the critical period of ocular growth and development in childhood, early and proactive intervention is essential to reduce the lifetime risk of sight-threatening complications.

The development of pediatric myopia has been largely attributed to rapid urbanization, reduced outdoor light exposure during childhood, and sustained near-work activities associated with modern educational systems. Sustained near work combined with insufficient exposure to distant visual targets may trigger a cascade of biochemical changes within the eye, resulting in reduced choroidal blood perfusion and a subsequent decrease in choroidal thickness [5,6,7]. Impaired choroidal perfusion may further compromise oxygen delivery to the sclera, thereby promoting scleral hypoxia [8]. Under hypoxic conditions, scleral fibroblasts downregulate type I collagen synthesis while upregulating matrix metalloproteinase expression, resulting in scleral extracellular matrix degradation [9,10]. Consequently, the sclera becomes biomechanically weakened and more susceptible to axial elongation under normal intraocular pressure, ultimately contributing to the development and progression of myopia. Within this pathogenic cascade, choroidal thinning plays a critical role in the early pathogenesis of myopia.

Recently, repeated low-level red-light (RLRL) therapy has emerged as a promising nonpharmacological intervention for controlling myopia progression in children. Red-light stimulation may enhance choroidal vasodilation and improve microcirculation, thereby promoting choroidal thickening [11]. An increase in choroidal thickness may anteriorly displace the retina, reducing hyperopic defocus, a key stimulus for axial elongation [12]. Therefore, RLRL-induced choroidal thickening may contribute to slowing axial length growth and myopia progression.

To date, only a limited number of meta-analyses have specifically examined changes in choroidal thickness following RLRL therapy based on randomized controlled trials (RCTs) [13,14]. Furthermore, several additional RCTs have been published in recent years, substantially expanding the available body of evidence [15,16]. Consequently, an updated and comprehensive synthesis of the current literature is warranted to generate more precise evaluations of the effects of RLRL therapy on choroidal thickness and to strengthen the existing evidence base. In addition, the potential differential effects of RLRL across different stages of refractive development, particularly between children with premyopia and those with established myopia, remain unclear. Therefore, we conducted a systematic review and meta-analysis of RCTs to quantitatively evaluate the impact of RLRL therapy on choroidal thickness and to perform prespecified subgroup analyses according to refractive status, thereby providing a more comprehensive assessment of its therapeutic effects in children with premyopia and myopia.

## 2. Materials and Methods

### 2.1. Search Strategy

This systematic review and meta-analysis was conducted in accordance with the Preferred Reporting Items for Systematic Reviews and Meta-Analyses (PRISMA) 2020 statement [17]. A comprehensive literature search was conducted in PubMed, Embase, and Cochrane Central Register of Controlled Trials, from database inception through 29 September 2026. The search strategy is described in Supplementary Table S1. Initially, titles and abstracts were independently screened to identify potentially eligible studies. Full-text articles were then assessed according to the predefined inclusion and exclusion criteria. In addition, the reference lists of all eligible studies and relevant articles were manually searched to identify additional potentially eligible publications. The study protocol was prospectively registered in the International Prospective Register of Systematic Reviews (PROSPERO; registration number CRD420261381602).

### 2.2. Eligibility Criteria

Two reviewers (Y.-Y.C. and H.-C.Y.) independently screened studies for eligibility. Only RCTs evaluating changes in subfoveal choroidal thickness (SFCT) following RLRL therapy were included. Nonrandomized studies, observational studies, retrospective studies, review articles, conference abstracts, and animal experiments were excluded to ensure a high level of evidence and minimize the risk of bias. In this review, studies enrolling participants younger than 18 years with clinically diagnosed myopia or premyopia were included. The intervention consisted of RLRL therapy administered twice daily via a dedicated desktop device emitting 650 nm red light. Eligible control groups included participants receiving standard care (e.g., single-vision spectacles) or sham treatment in placebo-controlled trials.

### 2.3. Data Extraction

The following study characteristics were collected: first author, publication year, sample size, follow-up duration, participant age, and SFCT measured in micrometers (μm). Continuous variables are presented as means ± standard deviations or medians with 95% confidence intervals (CIs) whenever available.

### 2.4. Quality Assessment

The methodological quality and risk of bias of the included studies were independently assessed by two reviewers (Y.-Y.C. and H.-C.Y.) using the Cochrane Risk of Bias 2 (RoB 2) tool. This tool is widely applied in randomized studies and assesses study quality across five domains: (1) bias arising from the randomization process, (2) bias due to deviations from intended interventions, (3) bias due to missing outcome data, (4) bias in the measurement of the outcome, and (5) bias in the selection of the reported results. Each study was classified as having a low risk of bias, some concerns, or a high risk of bias, and the overall risk of bias was judged according to the RoB 2 guidance. Any disagreements were resolved through discussion, and unresolved discrepancies were adjudicated by a third reviewer (Y.-F.Y.).

### 2.5. Statistical Analysis

Data were extracted independently by two reviewers (Y.-Y.C. and H.-C.Y.). The prespecified efficacy outcome for quantitative synthesis was the change in SFCT, assessed at standardized follow-up intervals of 1, 3, 6, 9, and 12 months. Statistical analyses were conducted using the Comprehensive Meta-Analysis software (version 4.0; Biostat, Englewood, NJ, USA). Changes from baseline at each follow-up time point were analyzed as continuous outcomes. For each outcome, the difference in mean change between the RLRL and control groups was calculated. The resulting mean differences (MDs) were pooled using random-effects models to estimate the overall treatment effects, with corresponding 95% CIs. A two-sided *p*-value of less than 0.05 was considered statistically significant. Statistical heterogeneity was assessed using the *I*^2^ statistic, with values of 50% or more considered indicative of substantial heterogeneity. Potential publication bias was assessed by visually inspecting funnel plots and Egger’s regression test to detect potential small-study effects and selective reporting. Sensitivity analyses were performed using a leave-one-out approach by sequentially excluding each individual study to evaluate the robustness and stability of the pooled estimates.

## 3. Results

### 3.1. Study Selection

**Figure 1:**
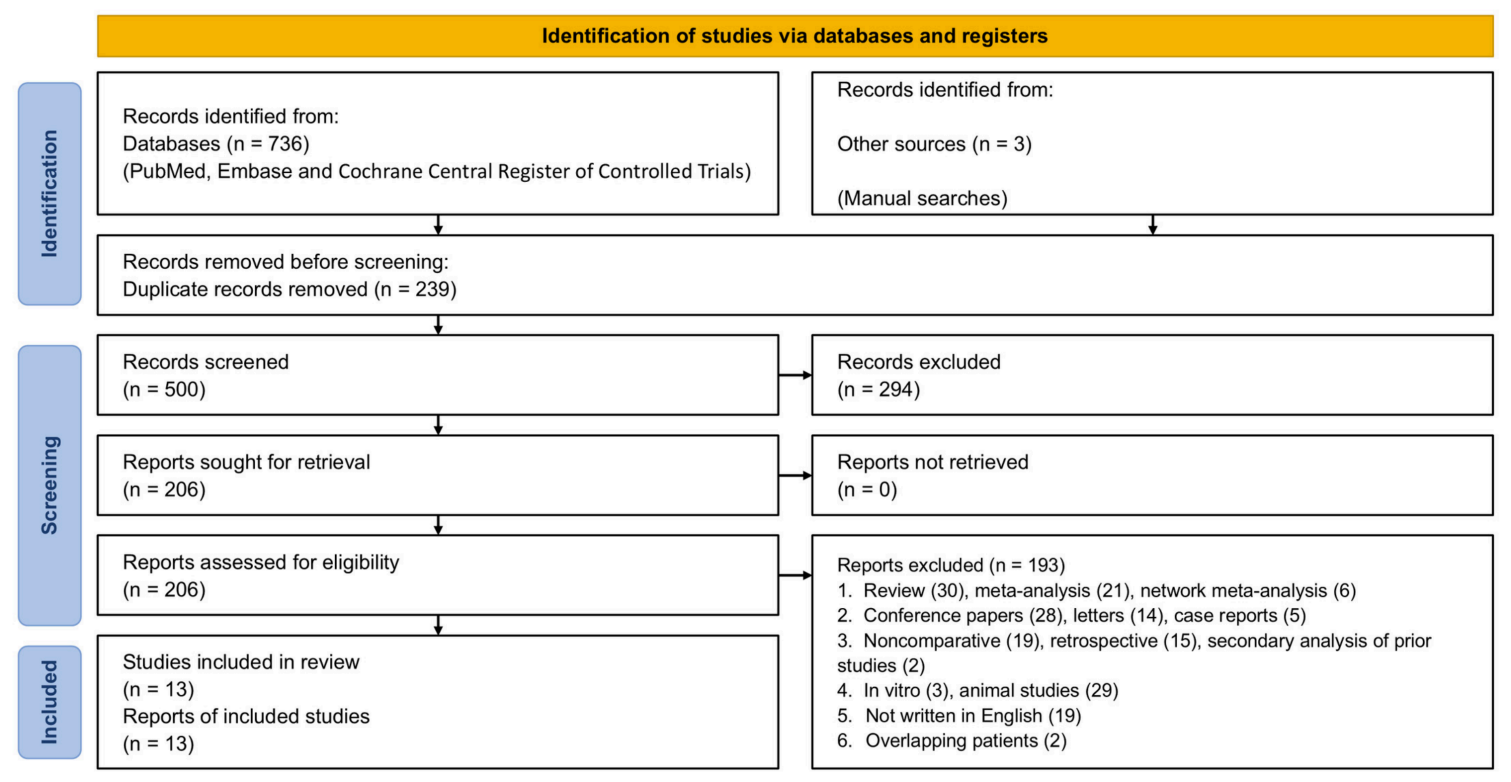
Study retrieval process according to the Preferred Reporting Items for Systematic Reviews and Meta-Analyses (PRISMA) 2020 statement.

The study selection process is illustrated in the PRISMA 2000 flow diagram (Figure 1), and the completed PRISMA checklist is provided in Supplementary Table S2 [17]. The initial database search identified 736 records from PubMed, Embase, and the Cochrane Central Register of Controlled Trials, and an additional three records were retrieved through manual searches. After removing 239 duplicate records, 500 unique studies were screened by title and abstract, resulting in the exclusion of 294 irrelevant articles. The full texts of the remaining 206 studies were subsequently assessed for eligibility.

Of these, 193 articles were excluded for the following reasons: review articles (n = 30), meta-analyses (*n* = 21), network meta-analyses (*n* = 6), conference papers (*n* = 28), letters (*n* = 14), case reports (*n* = 5), noncomparative studies (*n* = 19), retrospective studies (*n* = 15), secondary analyses of previously published studies (*n* = 2), in vitro studies (*n* = 3), animal studies (*n* = 29), studies with overlapping patients (*n* = 2) [15,16], and articles not published in English (*n* = 19). Ultimately, 13 high-quality RCTs, comprising 1974 pediatric participants, met the eligibility criteria and were included in the qualitative synthesis [18,19,20,21,22,23,24,25,26,27,28,29,30].

### 3.2. Characteristics of the Included Studies

The characteristics and baseline clinical data of the included studies are summarized in Table 1. The included studies enrolled children with a broad spectrum of baseline refractive statuses, ranging from premyopia to high myopia. Three studies exclusively included children with premyopia, defined as a spherical equivalent refraction greater than −0.5 D [28,29,30]. This wide distribution of baseline refractive statuses enabled prespecified subgroup analyses comparing children with premyopia and those with established myopia. The baseline SFCT ranged from approximately 215 to 317 μm across the included studies, while the follow-up durations ranged from 6 to 12 months.

Both Cao K 2024 [24] and Tian L 2023 [28] had the same trial ID and recruitment dates, indicating that they were derived from the same study population. However, for Cao K 2024 [24], we included only participants with myopia, whereas for Tian L 2023 [28], we included only non-myopic participants. Therefore, the populations included from these two studies were mutually exclusive, allowing both studies to be included independently in our meta-analysis. Regarding the Xiong F 2021 study [25], there were three groups: an orthokeratology (OK) lens-alone group, an RLRL-alone group, and a single-vision spectacle (SVS) control group. We included only the RLRL and SVS control groups in our statistical analyses; therefore, participants receiving OK lens treatment did not contribute to the pooled analyses and would not have confounded the results. Regarding the Zhou W 2024 study [23], there were also three groups: an RLRL 0.37 mW group, an RLRL 0.69 mW group, and a control group. We included only the RLRL 0.37 mW group and the control group in our statistical analyses. The 0.37 mW group was selected because its power level was closer to the commonly used RLRL power of 0.29 mW for myopia treatment.

### 3.3. Risk-of-Bias Assessment

**Figure 2:**
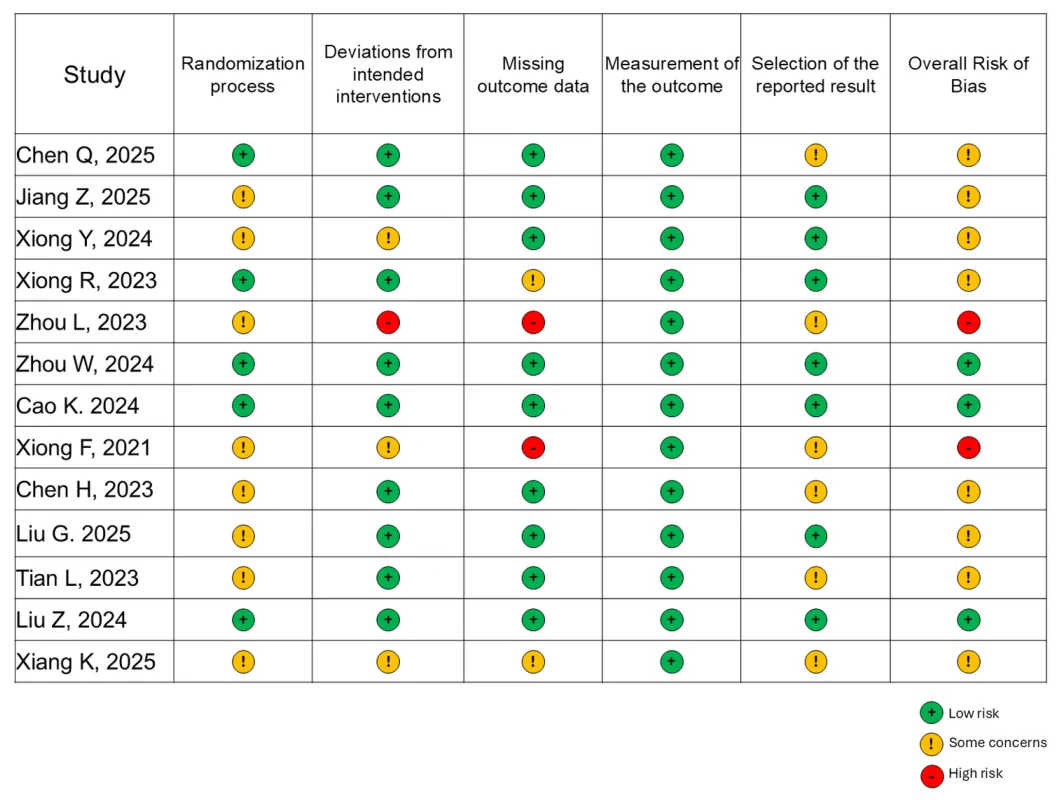
Risk-of-bias assessment of the included studies using the Cochrane Risk of Bias 2 (RoB2) tool [18,19,20,21,22,23,24,25,26,27,28,29,30].

The results of the risk-of-bias assessment are presented in Figure 2. Of the 15 included studies, five were judged to have a low risk of bias, eight were judged to have some concerns, and two were judged to have a high risk of bias. The measurement of the outcome domain was rated as a low risk of bias for all included studies. Some concerns were most commonly identified in the randomization process and selection of the reported results. Overall, two studies were classified as having a high risk of bias [21,24], mainly because of missing outcome data. In addition, one of these studies was judged to have a high risk of bias due to deviations from the intended interventions [21].

### 3.4. Outcome Assessment

**Figure 3:**
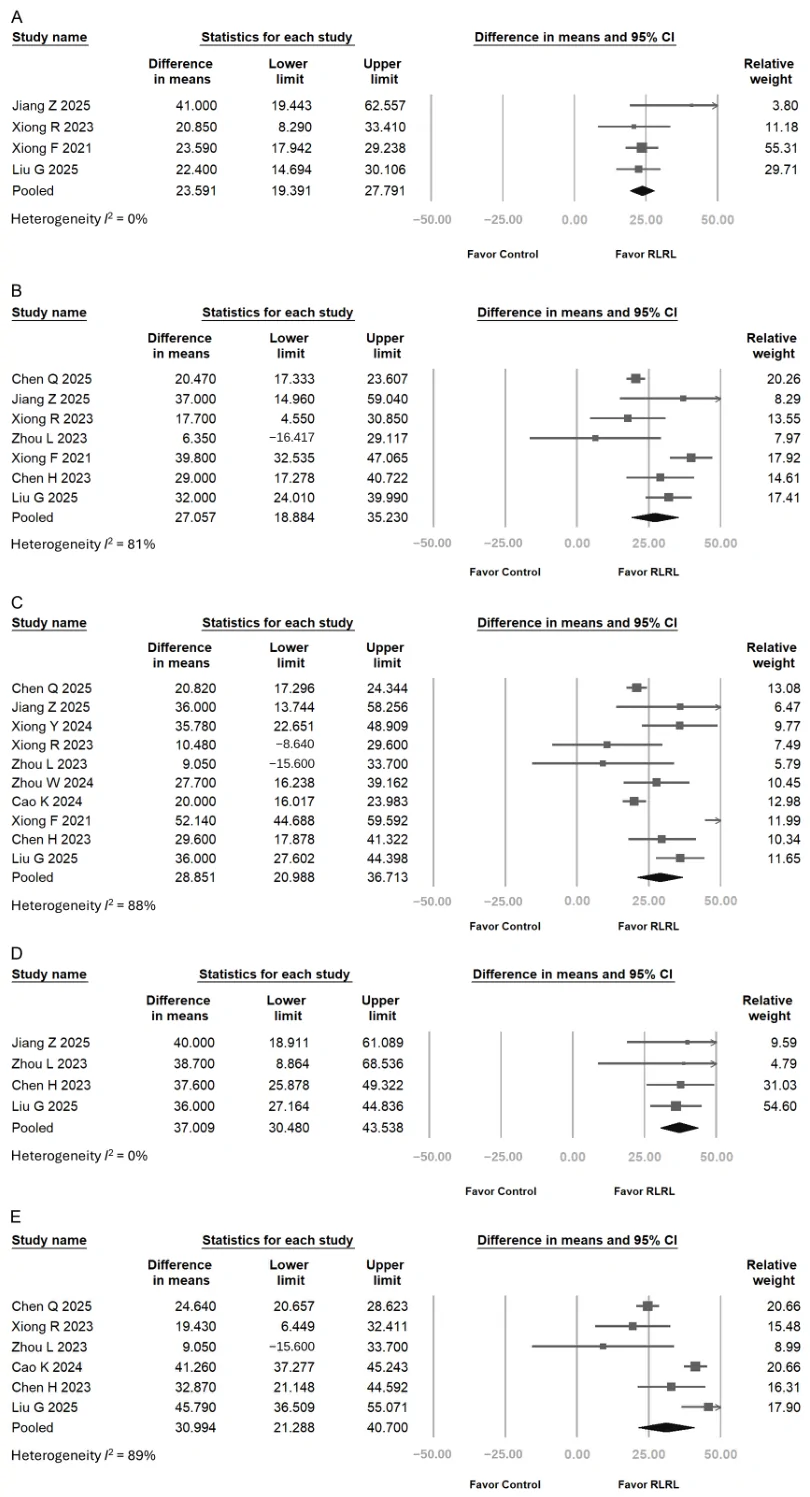
Forest plots of pooled changes in subfoveal choroidal thickness (SFCT) in children with myopia treated with repeated low-level red-light (RLRL) therapy compared with the control group. (**A**) 1 month, (**B**) 3 months, (**C**) 6 months, (**D**) 9 months, and (**E**) 12 months [18,19,20,21,22,23,24,25,26,27].

The data for the SFCT change at different time points across the included studies are summarized in Supplementary Table S3 [18,19,20,21,22,23,24,25,26,27,28,29,30]. Figure 3 shows that RLRL therapy was associated with a significantly greater increase in SFCT than the control group at all the evaluated time points. The pooled mean differences (MDs) were 23.59 μm (95% CI, 19.39–27.79; *I*^2^ = 0%) at 1 month; 27.06 μm (95% CI, 18.88–35.23; *I*^2^ = 81%) at 3 months; 28.85 μm (95% CI, 20.99–36.71; *I*^2^ = 88%) at 6 months; 37.01 μm (95% CI, 30.48–43.54; *I*^2^ = 0%) at 9 months; and 30.99 μm (95% CI, 21.29–40.70; *I*^2^ = 89%) at 12 months. Heterogeneity was absent at 1 and 9 months but substantial at 3, 6, and 12 months. Overall, RLRL therapy was associated with significantly greater SFCT thickening than the control intervention across all follow-up time points.

**Figure 4:**
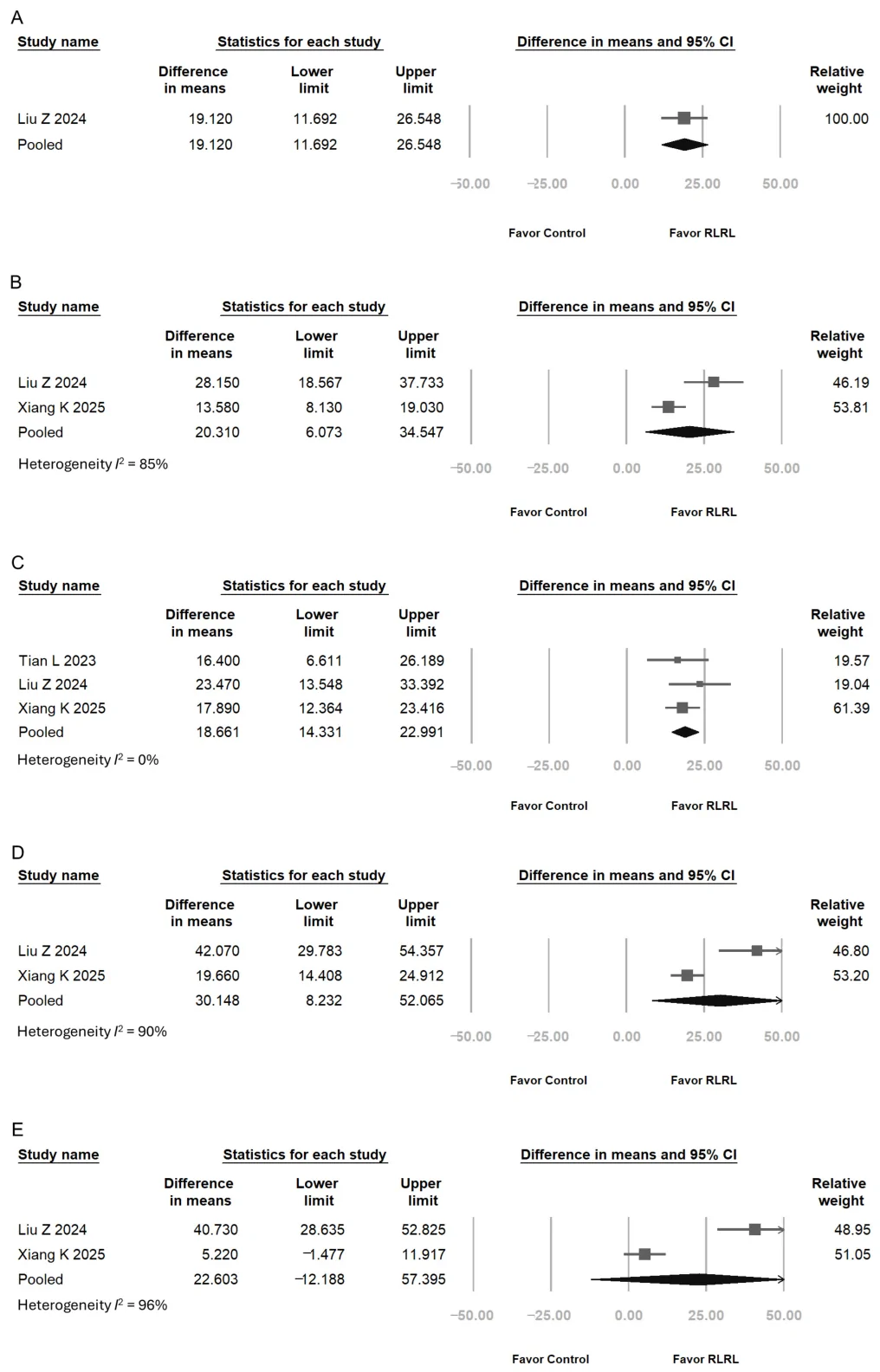
Forest plot of pooled changes in subfoveal choroidal thickness (SFCT) in children with premyopia treated with repeated low-level red-light (RLRL) therapy compared with the control group. (**A**) 1 month, (**B**) 3 months, (**C**) 6 months, (**D**) 9 months, and (**E**) 12 months [28,29,30].

Figure 4 presents the pooled effects of RLRL therapy on SFCT in children with premyopia compared with those in the control group. A significant increase in SFCT was observed as early as 1 month after treatment initiation (MD = 19.12 μm, 95% CI: 11.69–26.55). The treatment effect remained significant at 3 months (MD = 20.31 μm, 95% CI: 6.07–34.55), 6 months (MD = 18.66 μm, 95% CI: 14.33–22.99), and 9 months (MD = 30.15 μm, 95% CI: 8.23–52.07). At 12 months, the pooled estimate continued to favor RLRL therapy (MD = 22.60 μm, 95% CI: −12.19 to 57.40); however, the between-group difference was no longer statistically significant. Overall, RLRL therapy was associated with greater SFCT thickening than the control intervention throughout follow-up, except at 12 months.

### 3.5. Heterogeneity and Publication Bias

**Figure 5:**
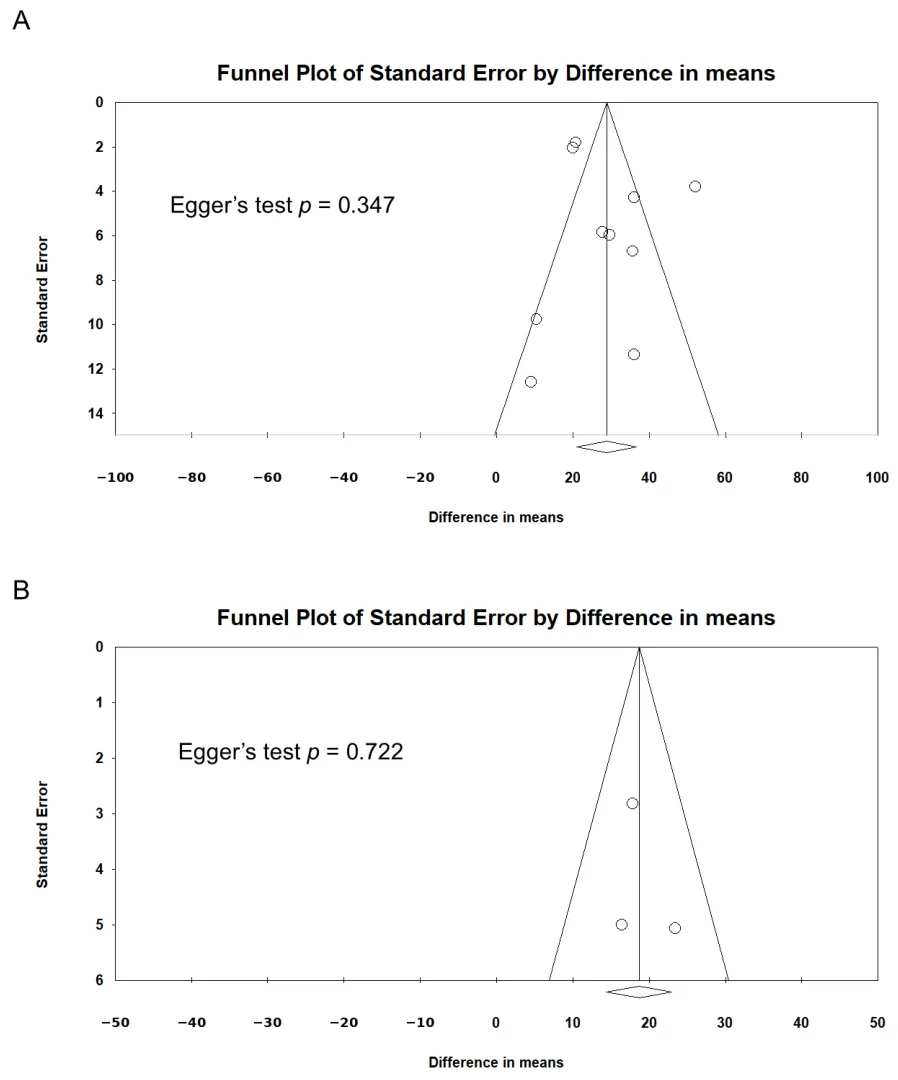
Funnel plots assessing publication bias using Egger’s regression test. (**A**) Subfoveal choroidal thickness (SFCT) change in children with myopia (*p* = 0.347). (**B**) SFCT change in children with premyopia (*p* = 0.722).

Most analyses demonstrated substantial heterogeneity, with *I*^2^ statistic ≥ 50%. Funnel plots assessing potential publication bias are presented in Figure 5. Visual inspection revealed no apparent asymmetry in either the myopia (Figure 5A) or premyopia (Figure 5B) subgroups. Furthermore, Egger’s regression tests were not statistically significant in any analysis (*p* ≥ 0.05), indicating no evidence of substantial publication bias.

### 3.6. Sensitivity Analysis

Leave-one-out sensitivity analyses demonstrated that the sequential exclusion of individual studies did not materially alter the pooled effect estimates or their statistical significance, supporting the stability and robustness of the overall findings.

Given that the Tian L 2023 study [28] included a heterogeneous non-myopic population (−0.50 D < SER ≤ +3.00 D), rather than a strictly defined premyopic population, we additionally performed a sensitivity analysis excluding this study. The exclusion of Tian L 2023 [28] did not alter the statistical significance of the pooled effect, indicating that the overall findings were not driven by the inclusion of this heterogeneous population.

We further excluded Zhou L 2023 [22] and Xiong F 2021 [25] because these studies were assessed as having a high risk of bias using the RoB 2 tool. Excluding either study individually or both studies simultaneously did not alter the statistical significance of the pooled effect, indicating that the overall findings were not driven by the inclusion of studies with a high risk of bias.

### 3.7. GRADE Summary of Findings

**Figure 6:**
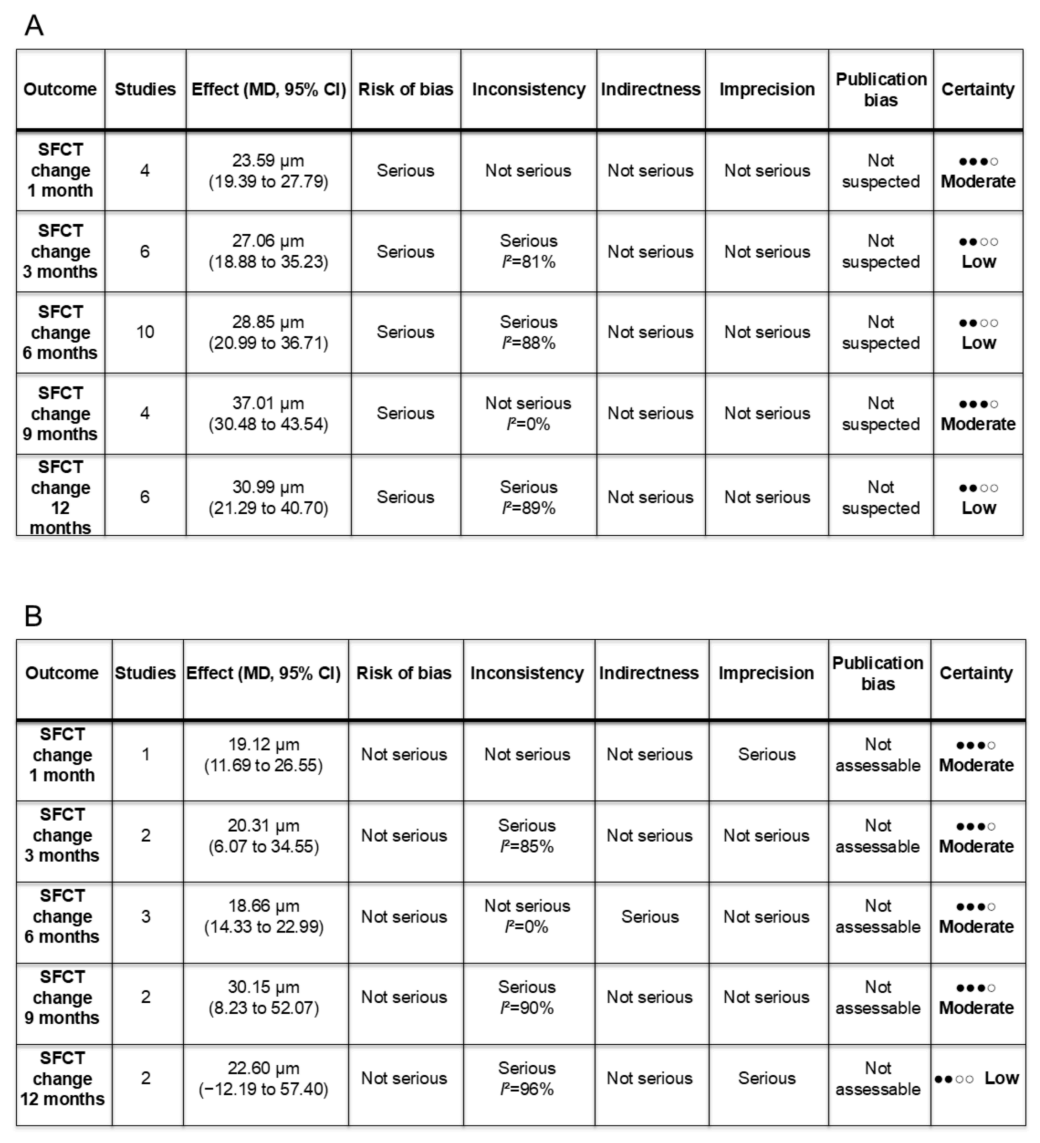
GRADE summary of findings for subfoveal choroidal thickness in children with myopia and premyopia. (**A**) Children with myopia. (**B**) Children with premyopia. GRADE, Grading of Recommendations Assessment, Development and Evaluation; MD, mean difference; SFCT, subfoveal choroidal thickness; CI, confidence interval.

The certainty of evidence for changes in SFCT following RLRL therapy is summarized in Figure 6. Among children with myopia, the certainty of evidence was moderate at 1 and 9 months and low at 3, 6, and 12 months. The certainty was downgraded primarily because of concerns regarding the risk of bias and, at 3, 6, and 12 months, substantial between-study heterogeneity (I^2^ = 81%, 88%, and 89%, respectively). Among children with premyopia, the certainty of evidence was moderate at 1, 3, 6, and 9 months and low at 12 months. Downgrading was mainly attributable to imprecision at 1 and 12 months; inconsistency at 3, 9, and 12 months; and indirectness at 6 months. Publication bias was not suspected for the myopia analyses but was not assessable for the premyopia analyses because of the limited number of studies.

## 4. Discussion

In this systematic review and meta-analysis, we synthesized evidence from 13 RCTs involving 1974 children to comprehensively evaluate the effects of RLRL therapy on SFCT. The results demonstrated that RLRL therapy consistently induced significant choroidal thickening in children with myopia throughout the 12-month follow-up period. Comparable findings were observed in children with premyopia, with significant increases in SFCT from 1 to 9 months; however, the pooled effect at 12 months was no longer statistically significant. Collectively, these findings indicate that choroidal thickening represents an early and sustained structural response to RLRL therapy and suggest a potential association between choroidal remodeling and the myopia-controlling effects of RLRL.

The present findings are consistent with the current understanding of the mechanisms underlying photobiomodulation. RLRL therapy delivers 650 nm red light, which is primarily absorbed by mitochondrial cytochrome c oxidase, thereby enhancing mitochondrial respiration, increasing adenosine triphosphate production, and promoting the transient release of nitric oxide [31,32,33,34]. These biochemical responses promote vasodilation and enhance choroidal blood perfusion, thereby increasing oxygen and nutrient delivery to the outer retina and sclera. Enhanced choroidal perfusion is thought to increase vascular permeability and intrachoroidal blood volume, leading to measurable increases in SFCT. The consistent thickening observed across nearly all follow-up time points in the present meta-analysis supports the hypothesis that RLRL induces sustained choroidal remodeling rather than merely eliciting a transient physiological response. Such structural changes may alleviate scleral hypoxia, improve metabolic support to the posterior pole, and reduce the biomechanical stimulus for axial elongation [31]. Therefore, the persistent increase in SFCT observed following RLRL therapy provides structural evidence supporting a plausible biological mechanism through which RLRL may slow myopia progression.

An important strength of the present study is the separate evaluation of children with premyopia and those with established myopia. Our subgroup analyses demonstrated that a significant increase in SFCT thickening also occurred in children with premyopia, indicating that the biological response to RLRL therapy is initiated before the onset of clinically significant myopia. Although the pooled effect at 12 months did not reach statistical significance, this finding should be interpreted with caution because only two studies contributed to the analysis at this time point, substantially limiting statistical power. Nevertheless, the significant increases in SFCT observed during the first 9 months indicate that RLRL therapy can induce choroidal remodeling during the earliest stages of refractive development. These findings imply that intervention during the premyopic stage may provide an opportunity to modify the biological trajectory of ocular growth before irreversible axial elongation is established. Consequently, early RLRL intervention may have important implications for myopia prevention.

In children with myopia, significant increases in SFCT were observed as early as 1 month after treatment initiation and were sustained throughout the 12-month follow-up period. This rapid structural response suggests that the choroid is among the earliest ocular tissues to respond to RLRL therapy. Clinically, optical coherence tomography enables non-invasive, readily available, and highly reproducible measurement of SFCT [35,36]. Future prospective studies should investigate whether early increases in SFCT predict long-term treatment success and whether the magnitude of choroidal thickening correlates with subsequent reductions in axial elongation.

Another important strength of this study should be acknowledged. By including only RCTs, we minimized the risk of selection bias and provided a high level of evidence. Furthermore, this review incorporates the most recently published RCTs, substantially expanding the available evidence base compared with previous meta-analyses.

This study also has several limitations. First, substantial statistical heterogeneity was observed across most pooled analyses. Variations in baseline refractive status, participant age, treatment duration, and study protocols may have contributed to the observed heterogeneity. Second, although all the included studies were RCTs, several were judged to have some concerns regarding the randomization process or selective reporting, and two studies were classified as having a high overall risk of bias. Third, relatively few studies enrolled children with premyopia, with only one study contributing to the 1-month analysis and only two to three studies contributing at subsequent time points. The limited number of studies reduces the precision of the pooled estimates and precludes the reliable assessment of publication bias in this subgroup. Finally, nearly all the included studies were conducted in East Asian populations, where the prevalence of myopia is particularly high, which may limit the generalizability of our findings to other ethnic groups and geographic regions. However, sensitivity analyses confirmed the robustness of the pooled estimates. Publication bias was not evident, and the overall methodological quality of the included studies was generally acceptable, supporting the reliability of the present findings.

## 5. Conclusions

In conclusion, this systematic review and meta-analysis demonstrates that RLRL therapy consistently increases SFCT in children with myopia and induces early beneficial structural changes in children with premyopia. These findings demonstrate that choroidal thickening is a consistent structural response to RLRL therapy in children with myopia and premyopia. Nevertheless, future multicenter RCTs with larger sample sizes and longer follow-up periods are needed to confirm the long-term durability of these effects and to determine whether early choroidal thickening is associated with sustained myopia control.

## Figures and Tables

**Table 1:** Characteristics of the studies enrolled in the meta-analysis.

First Author, Year	Inclusion Criteria of SER	Groups	Num	Age (Mean ± SD)	Baseline SFCT, µm (Mean ± SD)	Follow-Up Duration	Trial ID	Recruitment Dates
Chen Q, 2025 [18]	−6.0 D ≤ SER ≤ −0.5 D	RLRL	156	9.81 ± 1.60	245.51 ± 38.05	12 months	ChiCTR2100042836	February–March 2021
		Control	144	9.70 ± 1.76	246.21 ± 41.07			
Jiang Z, 2025 [19]	−5.0 D ≤ SER ≤ −1.0 D	RLRL	35	11.32 ± 2.05	244.79 ± 53.11	9 months	NR	NR
		Control	35	11.37 ± 2.08	244.94 ± 45.25			
Xiong Y, 2024 [20]	−6.0 D ≤ SER ≤ −0.5 D	RLRL	36	8.83 ± 2.06	251.83 ± 65.27	6 months	NR	March–June 2022
		Control	37	9.00 ± 2.00	274.76 ± 63.79			
Xiong R, 2023 [21]	−5.0 D ≤ SER ≤ −1.0 D	RLRL	60	10.52 ± 1.53	215.31 ± 58.52	12 months	NCT04073238	NR
		Control	60	10.37 ± 1.61	215.50 ± 57.71			
Zhou L, 2023 [22]	−8.0 D ≤ SER ≤ −0.5 D	RLRL	24	10.17 ± 1.55	259.32 ± 54.68	12 months	ChiCTR2100043619	NR
		Control	19	9.00 ± 1.20	239.77 ± 35.03			
Zhou W, 2024 [23]	SER ≤ −0.5 D	RLRL 0.37 mW	43	8.51 ± 1.51	255.09 ± 55.76	6 months	NR	April–May 2022
		Control	43	8.83 ± 1.53	267.58 ± 54.86			
Cao K, 2024 [24]	−6.0 D ≤ SER ≤ −0.5 D ^†^	RLRL	112	9.1 ± 2.0	289 ± 49	12 months	ChiCTR2200058963	12 August–3 September 2021
		Control	112	9.0 ± 1.9	291 ± 47			
Xiong F, 2021 [25]	SER ≤ −0.5 D	RLRL	74	10.2 ± 2.4	288.61 ± 59.59	6 months	NR	NR
		Control	74	10.3 ± 2.0	286.81 ± 63.67			
Chen H, 2023 [26]	−8.0 D ≤ SER ≤ −0.75 D	RLRL	46	9.00 ± 1.90	259.00 ± 51.46	12 months	ChiCTR2100045250	NR
		Control	40	8.98 ± 1.92	273.08 ± 54.37			
Liu G, 2025 [27]	SER ≤ −6.0 D	RLRL	130	10.7 ± 1.7	249 ± 69	12 months	NR	NR
		Control	68	10.5 ± 1.6	261 ± 64			
Tian L, 2023 [28]	−0.5 D < SER ≤ +3.0 D ^‡^	RLRL	56	7.7 ± 1.1	308 (261, 357) *	6 months	ChiCTR2200058963	12 August–3 September 2021
		Control	56	7.9 ± 1.8	317 (296, 356) *			
Liu Z, 2024 [29]	−0.5 D < SER < +0.75 D	RLRL	47	8.98 ± 1.31	NA	12 months	ChiCTR2200062028	NR
		Control	47	8.95 ± 1.52	NA			
Xiang K, 2025 [30]	−0.5 D < SER ≤ +0.5 D	RLRL	231	8.27 ± 1.10	265.80 ± 64.71	12 months	NR	NR
		Control	232	8.32 ± 1.09	278.63 ± 55.98			

Num, number; SFCT, subfoveal choroidal thickness; RLRL, repeated low-level red light; NR = not reported; * median (interquartile range). ^†^ Included only participants with myopia. ^‡^ Included only non-myopic participants.

## Data Availability

The data from this study are available on request from the corresponding author.

## Supplementary Materials

The following supporting information can be downloaded at: https://www.mdpi.com/article/10.3390/jcm15207862/s1, Table S1: Searching Strategies; Table S2: PRISMA 2020 Checklist; Table S3: SFCT change at different time points.

## Abbreviations

The following abbreviations are used in this manuscript:

RLRL	Repeated low-level red light
RCTs	Randomized controlled trials (RCTs)
PRISMA	Preferred Reporting Items for Systematic Reviews and Meta-Analyses
SFCT	Subfoveal choroidal thickness
CIs	Confidence intervals
RoB 2	Risk of bias 2
MDs	Mean differences
