# Supplementary material for: Effects of Repeated Low-Level Red-Light Therapy on Subfoveal Choroidal Thickness in Children with Myopia: A Systematic Review and Meta-Analysis
Source: J Clin Med. 2026 Oct 12;15(20):7862. doi: 10.3390/jcm15207862 (PMC13706064; doi:10.3390/jcm15207862)
Supplement: Supplementary file 1 [file jcm-15-07862-s001.zip › Supplementary Table S1.pdf]

Supplementary Table S1. Searching Strategies

**Pubmed**	
(<br>"red light"[tiab]<br>OR "red-light"[tiab]<br>OR "red light therapy"[tiab]<br>OR "red-light therapy"[tiab]<br>OR "repeated low-level red light"[tiab]<br>OR "repeated low level red light"[tiab]<br>OR "low-level red light"[tiab]<br>OR "low level red light"[tiab]<br>OR "low-intensity red light"[tiab]<br>OR "low intensity red light"[tiab]<br>OR "low-power red light"[tiab]<br>OR "low power red light"[tiab]<br>OR RLRL[tiab]<br>)<br>AND<br>(<br>myopia[MeSH]<br>OR myopia[tiab]<br>OR myopic[tiab]<br>OR premyopia[tiab]<br>OR "pre-myopia"[tiab]<br>OR "myopia control"[tiab]<br>OR "myopia progression"[tiab]<br>)	237
**Embase**	
(<br>'red light':ti,ab<br>OR 'red-light':ti,ab<br>OR 'red light therapy':ti,ab<br>OR 'red-light therapy':ti,ab<br>OR 'repeated low-level red light':ti,ab<br>OR 'repeated low level red light':ti,ab<br>OR 'low-level red light':ti,ab<br>OR 'low level red light':ti,ab<br>OR 'low-intensity red light':ti,ab<br>OR 'low intensity red light':ti,ab<br>OR 'low-power red light':ti,ab	356

OR 'low power red light':ti,ab<br>OR rlrl:ti,ab<br>)<br>AND<br>(<br>'myopia'/exp<br>OR myopia:ti,ab<br>OR myopic:ti,ab<br>OR premyopia:ti,ab<br>OR 'pre-myopia':ti,ab<br>OR 'myopia control':ti,ab<br>OR 'myopia progression':ti,ab<br>)	
**Cochrane CENTRAL**	
(<br>myopia<br>OR myopic<br>OR premyopia<br>OR "pre-myopia"<br>OR "myopia control"<br>OR "myopia progression"<br>)<br>AND<br>(<br>"red light"<br>OR "red-light"<br>OR "red light therapy"<br>OR "red-light therapy"<br>OR "repeated low-level red light"<br>OR "repeated low level red light"<br>OR "low-level red light"<br>OR "low level red light"<br>OR "low-intensity red light"<br>OR "low intensity red light"<br>OR "low-power red light"<br>OR "low power red light"<br>OR RLRL<br>)	143
