# Supplementary material for: Effects of Repeated Low-Level Red-Light Therapy on Subfoveal Choroidal Thickness in Children with Myopia: A Systematic Review and Meta-Analysis
Source: J Clin Med. 2026 Oct 12;15(20):7862. doi: 10.3390/jcm15207862 (PMC13706064; doi:10.3390/jcm15207862)
Supplement: Supplementary file 1 [file jcm-15-07862-s001.zip › Supplementary Table S3.pdf]

Supplementary Table S3. SFCT change at different time points

	RLRL			Control		
	mean	SD	n	mean	SD	n
**SfChT 1 month**						
Jiang Z, 2025 [19]	-2	45	35	39	47	35
Xiong R, 2023 [21]	17.96	34.5	60	-2.89	35.69	60
Xiong F, 2021 [25]	23.23	24.7	74	-0.36	2.09	74
Liu G, 2025 [27]	17.4	25.61	130	-5	27.5	68
Liu Z, 2024 [29]	16.96	19.87	47	-2.16	16.74	47
**SfChT 3 months**						
Chen Q, 2025 [18]	19.54	15.69	156	-0.93	11.53	144
Jiang Z, 2025 [19]	32	49	35	-5	45	35
Xiong R, 2023 [21]	6.59	34.04	60	-11.11	39.27	60
Xiong F, 2021 [25]	31.58	31.72	74	-8.22	3.24	74
Chen H, 2023 [26]	24.8	33.1	46	-4.2	19.6	40
Liu G, 2025 [27]	20	27.1	130	-12	27.5	68
Liu Z, 2024 [29]	19.58	20.59	47	-8.57	26.45	47
Xiang K, 2025 [30]	17.22	21.95	55	3.64	17.65	232
**SfChT 6 months**						
Chen Q 2025 [18]	20.28	16.82	156	-0.54	14.06	144
Jiang Z, 2025 [19]	27	48	35	-9	47	35
Xiong Y, 2024 [20]	29.19	32.82	36	-6.59	23.82	37
Xiong R, 2023 [21]	-1.39	47.2	60	-11.87	59.01	60
Zhou L, 2023 [22]	29.65	50.34	24	20.6	24.14	19
Zhou W, 2024 [23]	22.63	35.43	43	-5.07	14.67	43
Cao, 2024 [24]	-8	6.2	168	-11	5.1	168
Xiong F, 2021 [25]	35.3	31.75	74	-16.84	7.85	74
Chen H, 2023 [26]	25.2	33.1	46	-4.4	19.6	40
Liu G, 2025 [27]	22	29.2	130	-14	27.5	68
Tian, 2023 [28]	9.7	28.9	56	-6.7	23.7	56
Liu Z, 2024 [29]	15.85	23.77	47	-7.62	25.29	47
Xiang K, 2025 [30]	10.94	24.25	55	-6.95	17.28	232
**SfChT 9 months**						
Jiang Z, 2025 [19]	28	46	35	-12	44	35
Chen H, 2023 [26]	24.8	33.1	46	-12.8	19.6	40
Liu G, 2025 [27]	20	31.4	130	-16	27.5	68
Liu Z, 2024 [29]	19.57	35.51	47	-22.50	24.21	47
Xiang K, 2025 [30]	11.92	21.79	55	-7.74	16.82	232

**SfChT 12 months**						
Chen Q, 2025 [18]	23.46	19.62	156	-1.18	15.07	144
Xiong R, 2023 [21]	7.34	34.89	60	-12.09	37.61	60
Zhou L, 2023 [22]	29.65	50.34	24	20.6	24.14	19
Cao, 2024 [24]	-20	6.2	168	-23	5.1	168
Chen H, 2023 [26]	21.57	33.1	46	-11.3	19.6	40
Liu G, 2025 [27]	21.02	33.59	130	-24.77	27.5	68
Liu Z, 2024 [29]	11.99	32.66	47	-28.74	26.89	47
Xiang K, 2025 [30]	-0.94	27.87	55	-6.16	21.42	232
